# Risk factors and prediction modeling for drug-resistant tuberculosis in Shenzhen: a 9-year retrospective study

**DOI:** 10.3389/fpubh.2026.1875521

**Published:** 2026-09-01

**Authors:** Jin Wang, Moru Chen, Xiaoliang Zha, Feng Ding, He Zhang, Shui-hua Lu, Xiangxiang Liu

**Affiliations:** 1 Shenzhen Third People’s Hospital, The Second Affiliated Hospital of Southern University of Science and Technology, Shenzhen, China; 2 National Clinical Research Center for Infectious Disease of Shenzhen, Shenzhen Third People’s Hospital, The Second Affiliated Hospital of Southern University of Science and Technology, Shenzhen, China

**Keywords:** drug-resistant tuberculosis, prediction model, receiver operating characteristic (ROC), risk factors, tuberculosis

## Abstract

**Background:**

Antimicrobial resistance in *Mycobacterium tuberculosis* (*M.tb*) poses a major threat to global tuberculosis (TB) control, limiting treatment options and increasing mortality. The prevalence and risk factors for drug-resistant TB (DR-TB) vary by socioeconomic and demographic context. Shenzhen, a rapidly developing megacity in China with a large migrant population, provides a critical setting for investigating this issue. This study aimed to analyze the DR-TB trends and risk factors in Shenzhen using a nine-year retrospective analysis.

**Methods:**

We retrospectively analyzed 16,466 TB patients managed at Shenzhen Third People’s Hospital from January 2017 and December 2025. Risk factors for DR-TB were identified using univariate and multivariate logistic regression. Based on these factors, we constructed four prediction models (demographic, demographic+clinical symptom, demographic+clinical symptom+diagnostic, and demographic+clinical symptom+diagnostic+treatment delay), and evaluated their performance using Receiver Operating Characteristic (ROC) curves and calibration curves.

**Results:**

The prevalence of DR-TB decreased from 11.36% in 2017 to 10.42% in 2019, dropped sharply to 7.9% in 2020–2021 (likely due to COVID-19 disruptions), rebounded to 14.63% in 2022 (above pre-pandemic levels), peaked in 2023, and then gradually declined after 2024. Multivariate logistic regression identified multiple factors significantly associated with drug resistance: gender (female), age (25–44, 45–64, ≥65 years), occupation (unemployed, family workers, business/service providers, office workers, students), HIV/AIDS comorbidity, clinical symptoms (cough, sputum production), ≥1 negative etiological test result, re-treatment status, concurrent extra pulmonary TB, time from symptom onset to first medical visit (28–42 days), and time from first visit to diagnosis (≥42 days). The AUC values for the four models were 0.571, 0.599, 0.760, and 0.787, respectively (all *p* < 0.001). Hosmer-Lemeshow *χ*^2^ values were 14.014, 13.402, 9.618 and 8.612, respectively (*p* > 0.05).

**Conclusion:**

We successfully developed a DR-TB risk prediction model with good discrimination and calibration. Early identification of risk factors is crucial for improving clinical awareness and management of DR-TB. Enhancing early diagnosis and treatment is essential for controlling the disease.

**Clinical trial registration:**

https://www.chictr.org.cn/showproj.html?proj=188950, Identifier [ChiCTR2300067585].

## 1 Background

Drug-resistant tuberculosis (DR-TB), particularly multidrug-resistant (MDR-TB) and extensively drug-resistant TB (XDR-TB), poses a major threat to global TB control (1). MDR-TB, resistant to at least isoniazid and rifampin, is a significant challenge, with an estimated 390,000 incident cases globally in 2024. While the annual number has plateaued since 2020, it remains a pressing concern (2). China bears a high TB burden, with an estimated 696,000 new cases in 2024, including approximately 28,000 MDR/RR-TB cases, accounting for 7.1% of the global total (2).

The emergence of DR-TB is driven by factors such as poor public awareness, diagnostic delays, inadequate treatment, and direct transmission of resistant strains (3). Within China, the magnitude and patterns of drug resistance vary considerably across regions due to vast geographical size, diverse demographics, and uneven economic development (4). Shenzhen, a rapidly growing megacity in southern China adjacent to Hong Kong, exemplifies this complexity. Its population has surged from 30,000 in 1979 to over 17 million, with migrants constituting more than 67% of residents (5, 6). Despite its economic prominence, Shenzhen reports a high TB incidence (43.94/100,000) (7). Among its migrant population, the estimated MDR/RR-TB prevalence is 17.87%, yet many cases remain undiagnosed and untreated (8). This unique demographic and geographic setting suggests that DR-TB in Shenzhen may have distinct epidemiological characteristics. However, the true scale and risk factors of DR-TB in the city have not been well characterized, hindering the development of effective control strategies.

To address this gap, we conducted a retrospective study (2017–2025) using data from Shenzhen Third People’s Hospital. We analyzed demographic, clinical, and diagnostic data to determine the prevalence trends and risk factors for DR-TB. The findings aim to inform targeted TB control strategies for Shenzhen and similar high-mobility urban centers.

## 2 Methods

### 2.1 Study design and data source

We performed a retrospective study using electronic medical records from Shenzhen Third People’s Hospital, a designated TB treatment center.

### 2.2 Data collection and study population

We analyzed de-identified data from patients diagnosed with TB between January 2017 and December 2025. Collected information included demographics, clinical symptoms, comorbidities, drug resistance profiles, treatment history, etiological diagnostic results (culture, smear, molecular testing), dates related to diagnosis, and drug susceptibility testing (DST) data.

TB diagnosis was confirmed based on a combination of clinical symptoms, chest computed tomography, and laboratory evidence (sputum smear, culture, or molecular test). For patients with multiple records, only the first complete entry was retained. Two trained researchers independently extracted the relevant data.

### 2.3 Definitions

Case definitions followed the World Health Organization consolidated guidelines (9) and Chinese national standards (WS 288-2017) (10):

- Drug-Susceptible TB (DS-TB): Bacteriologically confirmed or clinically diagnosed TB without resistance to rifampicin, isoniazid, or other first-line anti-TB drugs.
- Drug-Resistant TB (DR-TB): Bacteriologically confirmed or clinically diagnosed TB with resistance to at least one anti-TB drugs.
- Poly-resistant TB (PDR-TB): Resistance to more than one first-line anti-TB drug, but not simultaneously to both isoniazid and rifampicin.
- Mone-Resistant TB (MR-TB): Resistance to a single anti-TB drug.
- Rifampicin-Resistant TB (RR-TB): TB caused by strains resistant to rifampicin.
- Isoniazid Monoresistant TB (Hr-TB): TB caused by strains resistant to isoniazid but susceptible to rifampicin.
- Multidrug-Resistant TB (MDR-TB): TB caused by strains resistant to rifampicin and isoniazid.
- Extensively drug-resistant TB (XDR-TB): TB caused by strains resistant to rifampicin (and possibly also to isoniazid), and additionally resistant to at least one fluoroquinolone (levofloxacin or moxifloxacin) and at least one other Group A drug (bedaquiline or linezolid). Although WHO has updated the definition (11), the current system data has not yet been updated, therefore, this study employs the old definition. Moreover, we conducted sensitivity analysis using data from 2023–2025 without altering the existing conclusions.

### 2.4 Statistical analysis

All analyses were performed using SPSS software (version 26.0). Categorical variables are summarized as frequencies and percentages. Univariate screening of risk factors was performed using the chi-square test. Variables significant in univariate analysis were sequentially entered into multivariate logistic regression to construct four prediction models: demographic (Model 1), demographic + clinical symptom (Model 2), demographic + clinical symptom + diagnostic (Model 3), and demographic + clinical symptom + diagnostic + treatment delay (Model 4). Model performance was evaluated using specificity, sensitivity, ROC curves, area under the curve (AUC), Hosmer-Lemeshow goodness-of-fit test, and calibration curves. A two-sided *p* < 0.05 was considered statistically significant.

## 3 Results

### 3.1 General characteristics

A total of 16,466 TB patients were included after excluding 127 with nontuberculosis mycobacteria (NTM) infection. The majority patients had DS-TB (87.84%, *n* = 14,464), while 12.16% (*n* = 2,002) had DR-TB. Among DR-TB cases, RR-TB was most common (84.47%), followed by MR-TB (52.90%), MDR-TB (45.902%), Hr-TB (14.34%), XDR-TB (6.24%), and PDR-TB (1.15%).

The mean age was 42.64 ± 17.702 years, with most patients (71.95%) being young adults (25–64 years). Males accounted for 61.92%. Comorbidities included HIV/AIDS (0.12%), and diabetes (8.54%). Common symptoms were cough, sputum production, fever, hemoptysis, and chest pain. A history of previous TB treatment was present in 10.56% of TB patients (Table 1). Diagnostic methods included sputum smear (38.19%), culture (57.87%), and molecular biology (59.99%).

**Table 1:** Distribution of drug-resistant and drug-sensitive TB by demographic and clinical characteristics in Shenzhen, 2017–2025*.

Characteristic		Total TB*		DS-TB*		DR-TB *(*N* = 2002)											
		(*N* = 16,466)		(*N* = 14,464)		MR-TB* (*N* = 1,059)		PDR-TB* (*N* = 23)		RR-TB* (*N* = 1,691)		Hr-TB* (*N* = 287)		MDR-TB* (*N* = 919)		XDR-TB* (*N* = 125)	
		*n*	%	*n*	%	*n*	%	*n*	%	*n*	%	*n*	%	*n*	%	*n*	%
Gender	Male	10,195	61.92	8,910	61.60	683	64.49	10	43.48	1,094	64.70	177	61.67	588	63.98	65	52.00
	Female	6,271	38.08	5,554	38.40	376	35.51	13	56.52	597	35.30	110	38.33	331	36.02	60	48.00
Age (years)	<25	2,314	14.05	2064	14.27	145	13.69	3	13.04	207	12.24	42	14.63	104	11.32	11	8.80
	25–44	7,265	44.12	6,319	43.69	488	46.08	15	65.22	819	48.43	116	40.42	447	48.64	59	47.20
	45–64	4,582	27.83	3,963	27.40	328	30.97	4	17.39	521	30.81	91	31.71	284	30.90	43	34.40
	≥65	2,305	14.00	2,118	14.64	98	9.25	1	4.35	144	8.52	38	13.24	84	9.14	12	9.60
Mobility	Intra-urban	5,817	35.33	5,134	35.50	347	32.77	3	13.04	573	33.89	105	36.59	331	36.02	36	28.80
	Inter-city	2,872	17.44	2,537	17.54	181	17.09	1	4.35	270	15.97	61	21.25	150	16.32	16	12.80
	Inter provincial	7,777	47.23	6,793	46.96	531	50.14	19	82.61	848	50.15	121	42.16	438	47.66	73	58.40
Ethnicity	Han	16,246	98.66	14,270	98.66	1,044	98.58	23	100.00	1,669	98.70	283	98.61	908	98.80	123	98.40
	Others	220	1.34	194	1.34	15	1.42	0	0.00	22	1.30	4	1.39	11	1.20	2	1.60
Occupation	Unemployed	5,530	33.58	4,882	33.75	356	33.62	1	4.35	543	32.11	100	34.84	287	31.23	36	28.80
	Family workers	4,179	25.38	3,724	25.75	252	23.80	4	17.39	368	21.76	83	28.92	199	21.65	19	15.20
	Business service providers	2030	12.33	1705	11.79	157	14.83	4	17.39	293	17.33	30	10.45	166	18.06	28	22.40
	Retirees	777	4.72	693	4.79	38	3.59	4	17.39	72	4.26	11	3.83	45	4.90	5	4.00
	Office workers	3,008	18.27	2,590	17.91	220	20.77	10	43.48	358	21.17	49	17.07	187	20.35	33	26.40
	Students	858	5.21	793	5.48	32	3.02	0	0.00	50	2.96	14	4.88	32	3.48	2	1.60
	Others	84	0.51	77	0.53	4	0.38	0	0.00	7	0.41	0	0.00	3	0.33	2	1.60
Combined with HIV/AIDS	No	16,447	99.88	14,451	99.91	1,055	99.62	23	100.00	1,685	99.65	287	100.00	917	99.78	123	98.40
	Yes	19	0.12	13	0.09	4	0.38	0	0.00	6	0.35	0	0.00	2	0.22	2	1.60
Combined with Diabetes	No	15,060	91.46	13,212	91.34	959	90.56	23	100.00	1,572	92.96	253	88.15	866	94.23	115	92.00
	Yes	1,406	8.54	1,252	8.66	100	9.44	0	0.00	119	7.04	34	11.85	53	5.77	10	8.00
Close contacts of TB	No	16,457	99.95	14,457	99.95	1,057	99.81	23	100.00	1,689	99.88	287	100.00	919	100.00	125	100.00
	Yes	9	0.05	7	0.05	2	0.19	0	0.00	2	0.12	0	0.00	0	0.00	0	0.00
Cough	No	11,511	69.91	10,294	71.17	637	60.15	9	39.13	987	58.37	221	77.00	571	62.13	62	49.60
	Yes	4,955	30.09	4,170	28.83	422	39.85	14	60.87	704	41.63	66	23.00	348	37.87	63	50.40
Sputum production	No	13,958	84.77	12,417	85.85	824	77.81	12	52.17	1,277	75.52	250	87.11	703	76.50	81	64.80
	Yes	2,508	15.23	2047	14.15	235	22.19	11	47.83	414	24.48	37	12.89	216	23.50	44	35.20
Fever	No	14,829	90.06	13,047	90.20	930	87.82	21	91.30	1,496	88.47	267	93.03	833	90.64	108	86.40
	Yes	1,637	9.94	1,417	9.80	129	12.18	2	8.70	195	11.53	20	6.97	86	9.36	17	13.60
Hemoptysis	No	15,621	94.87	13,753	95.08	984	92.92	20	86.96	1,568	92.73	278	96.86	862	93.80	111	88.80
	Yes	845	5.13	711	4.92	75	7.08	3	13.04	123	7.27	9	3.14	57	6.20	14	11.20
Chest pain	No	15,672	95.18	13,783	95.29	979	92.45	22	95.65	1,583	93.61	283	98.61	887	96.52	119	95.20
	Yes	794	4.82	681	4.71	80	7.55	1	4.35	108	6.39	4	1.39	32	3.48	6	4.80
Other clinical symptoms	No	16,241	98.63	14,267	98.64	1,039	98.11	23	100.00	1,664	98.40	286	99.65	911	99.13	124	99.20
	Yes	223	1.35	195	1.35	20	1.89	0	0.00	27	1.60	1	0.35	8	0.87	1	0.80
Etiologic diagnosis results	Positive	13,303	80.79	11,329	78.33	1,038	98.02	23	100.00	1,671	98.82	280	97.56	913	99.35	124	99.20
	≥1 negative in sputum culture, smear, or molecular test	3,163	19.21	3,135	21.67	21	1.98	0	0.00	20	1.18	7	2.44	6	0.65	1	0.80
Treatment type	Newly treated patients	14,727	89.44	13,446	92.96	739	69.78	12	52.17	1,010	59.73	250	87.11	521	56.69	59	47.20
	Retreated patients	1739	10.56	1,018	7.04	320	30.22	11	47.83	681	40.27	37	12.89	398	43.31	66	52.80
Combined with EPTB infections	No	11,664	70.84	10,076	69.66	818	77.24	19	82.61	1,368	80.90	206	71.78	756	82.26	103	82.40
	Yes	4,802	29.16	4,388	30.34	241	22.76	4	17.39	323	19.10	81	28.22	163	17.74	22	17.60
Days between symptom onset and first medical	<14	7,196	43.70	6,472	44.75	436	41.17	15	65.22	584	34.54	130	45.30	278	30.25	38	30.40
	14–28	3,032	18.41	2,721	18.81	192	18.13	4	17.39	256	15.14	50	17.42	114	12.40	20	16.00
	28–42	2,344	14.24	1888	13.05	172	16.24	2	8.70	410	24.25	40	13.94	278	30.25	12	9.60
	≥42	3,894	23.65	3,383	23.39	259	24.46	2	8.70	441	26.08	67	23.34	249	27.09	55	44.00
Days between first medical visit and diagnosis	<14	2,227	13.52	2049	14.17	436	41.17	3	13.04	136	8.04	41	14.29	62	6.75	13	10.40
	14–28	4,260	25.87	3,900	26.96	192	18.13	6	26.09	291	17.21	61	21.25	103	11.21	14	11.20
	28–42	2,824	17.15	2,551	17.64	172	16.24	2	8.70	227	13.42	44	15.33	104	11.32	13	10.40
	≥42	7,155	43.45	5,964	41.23	259	24.46	12	52.17	1,037	61.32	141	49.13	650	70.73	85	68.00
Total		16,466	100.00	14,464	100.00	1,059	100.00	23	100.00	1,691	100.00	287	100.00	919	100.00	125	100.00

*TB, tuberculosis; EPTB, extra pulmonary tuberculosis; DS-TB, drug-sensitive tuberculosis; DR-TB, drug-resistant tuberculosis; MR-TB, mono-resistant tuberculosis; PDR-TB, poly-resistant tuberculosis; RR-TB, rifampin resistance tuberculosis; Hr-TB, isoniazid resistance tuberculosis; MDR-TB, multi drug-resistant tuberculosis; XDR-TB, extensively drug-resistant tuberculosis; HIV/AIDS, Human immunodeficiency virus/Acquired immunodeficiency syndrome.

**Figure 1:**
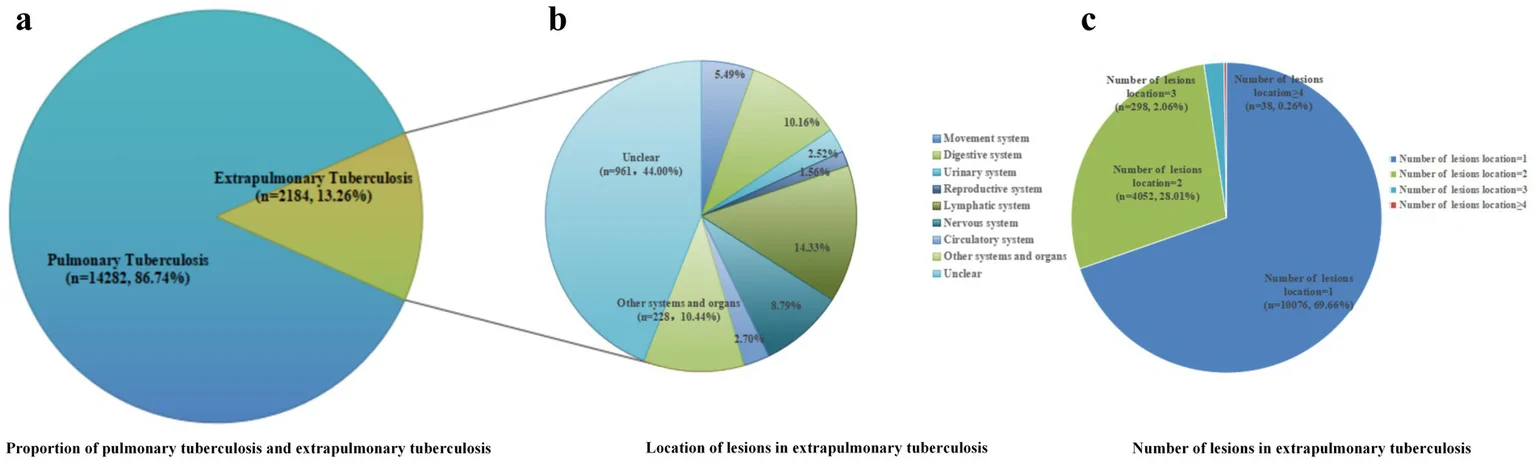
Characteristics of extrapulmonary tuberculosis (EPTB). **(a)** Proportion of patients with pulmonary TB only versus combined pulmonary and EPTB. **(b)** Distribution of the primary anatomical sites of EPTB lesions. **(c)** Number of distinct anatomical sites infected with *M. tuberculosis* per patient.

Extrapulmonary TB (EPTB) complications occurred in 13.26% (n = 2,184) of all TB patients (Figure 1a). Lymphatic EPTB was most frequent (14.33%), followed by digestive system involvement (10.16%) (Figure 1b). The number of anatomical sites infected varied: 69.66% having one site, 28.01% had two, 2.06% had three, and 0.26% had four or more (Figure 1c).

### 3.2 Trends in TB prevalence (2017–2025)

**Figure 2:**
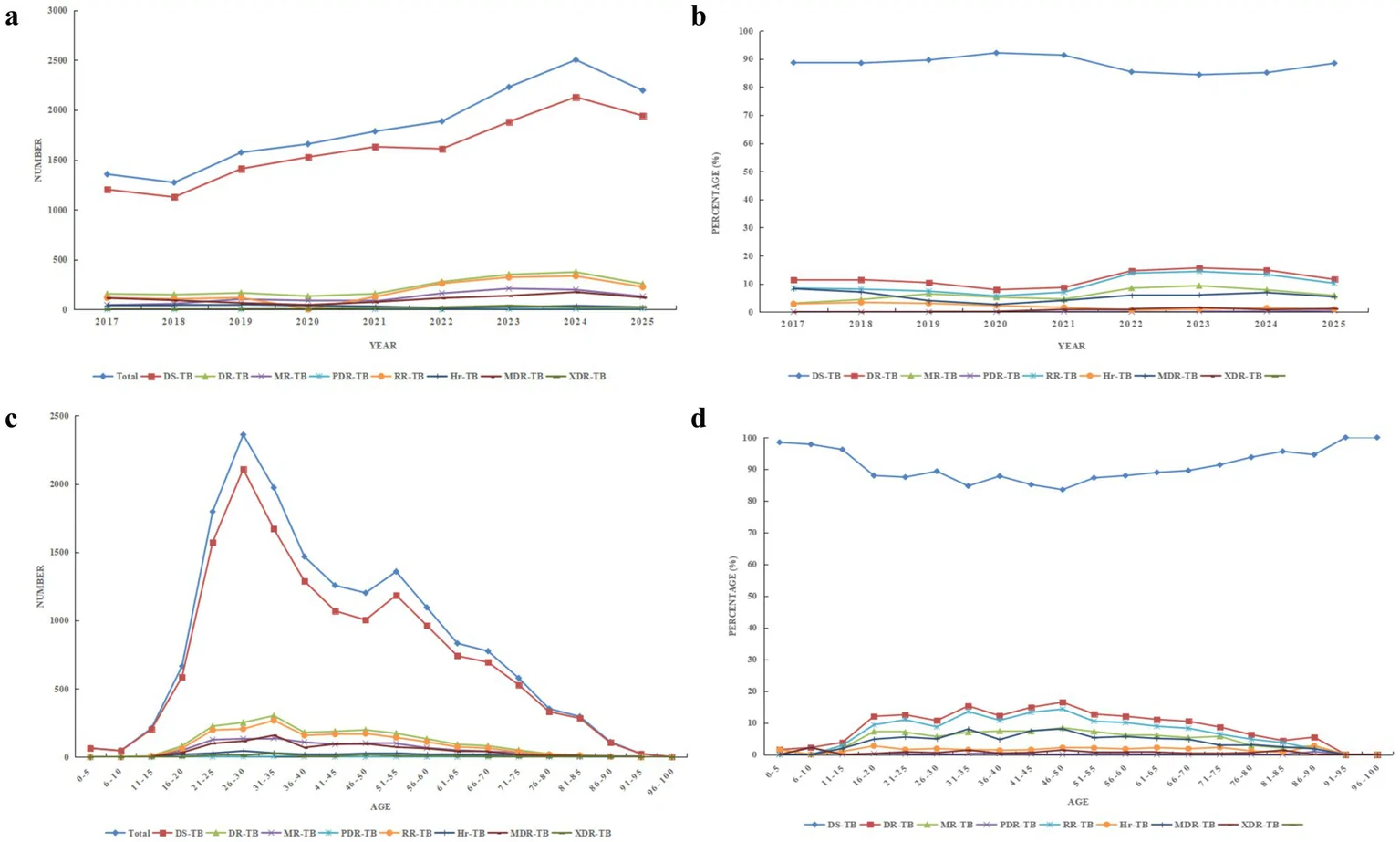
Epidemiological trends of tuberculosis (TB) in Shenzhen, China (2017–2025). **(a)** Number of patients with TB, DS-TB, DR-TB, MR-TB, PDR-TB, RR-TB, Hr-TB, MDR-TB, and XDR-TB from 2017 to 2025. **(b)** Prevalence of patients with DS-TB, DR-TB, MR-TB, PDR-TB, RR-TB, Hr-TB, MDR-TB, and XDR-TB from 2017 to 2025. **(c)** Number of patients with TB, DS-TB, DR-TB, MR-TB, PDR-TB, RR-TB, Hr-TB, MDR-TB, and XDR-TB in different age groups. **(d)** Prevalence of patients with DS-TB, DR-TB, MR-TB, PDR-TB, RR-TB, Hr-TB, MDR-TB, and XDR-TB in different age groups. TB, tuberculosis; DS-TB, drug-sensitive tuberculosis; DR-TB, drug-resistant tuberculosis; MR-TB, mono-resistant tuberculosis; PDR-TB, poly-resistant tuberculosis; RR-TB, rifampin resistance tuberculosis; Hr-TB, isoniazid resistance tuberculosis; MDR-TB, multidrug-resistant tuberculosis; XDR-TB, extensively drug-resistant tuberculosis.

From 2017 and 2025, total TB and DS-TB cases increased, while DR-TB cases remained stable in number (Figure 2a). The prevalence of DR-TB decreased from 11.36% in 2017 to 10.42% in 2019, dropped markedly to 7.9% in 2020–2021 (likely reflecting COVID-19-related healthcare disruptions), rebounded sharply to 14.63% in 2022 (above pre-pandemic levels), continued to rise in 2023, and then steadily declined after 2024. Trends for MR-TB, PDR-TB, RR-TB, Hr-TB, MDR-TB, and XDR-TB followed a similar pattern (Figure 2b).

Age distribution analysis showed that TB, DS-TB, DR-TB, and all resistance subtypes were primarily concentrated in the 15–65 age group. Cases numbers rose up to age 30, then declined, with a slight rebound at 51–55 years, and a continued decline after 55 (Figure 2c). The highest drug resistance rate (16.47%) occurred in the 46–50 age group, followed by 31–35 years (15.31%) (Figure 2d).

### 3.3 Univariate analysis of factors associated with DR-TB

Univariate analysis identified several factors significantly associated (*p* < 0.05) with drug resistance. These included gender, age, occupation, HIV/AIDS comorbidity, symptoms (cough, sputum production, hemoptysis), etiologic test results (culture, smear, and molecular), treatment history (new vs. retreatment), concurrent EPTB, and all diagnostic delay intervals (see Supplementary Table 1).

**Figure 3:**
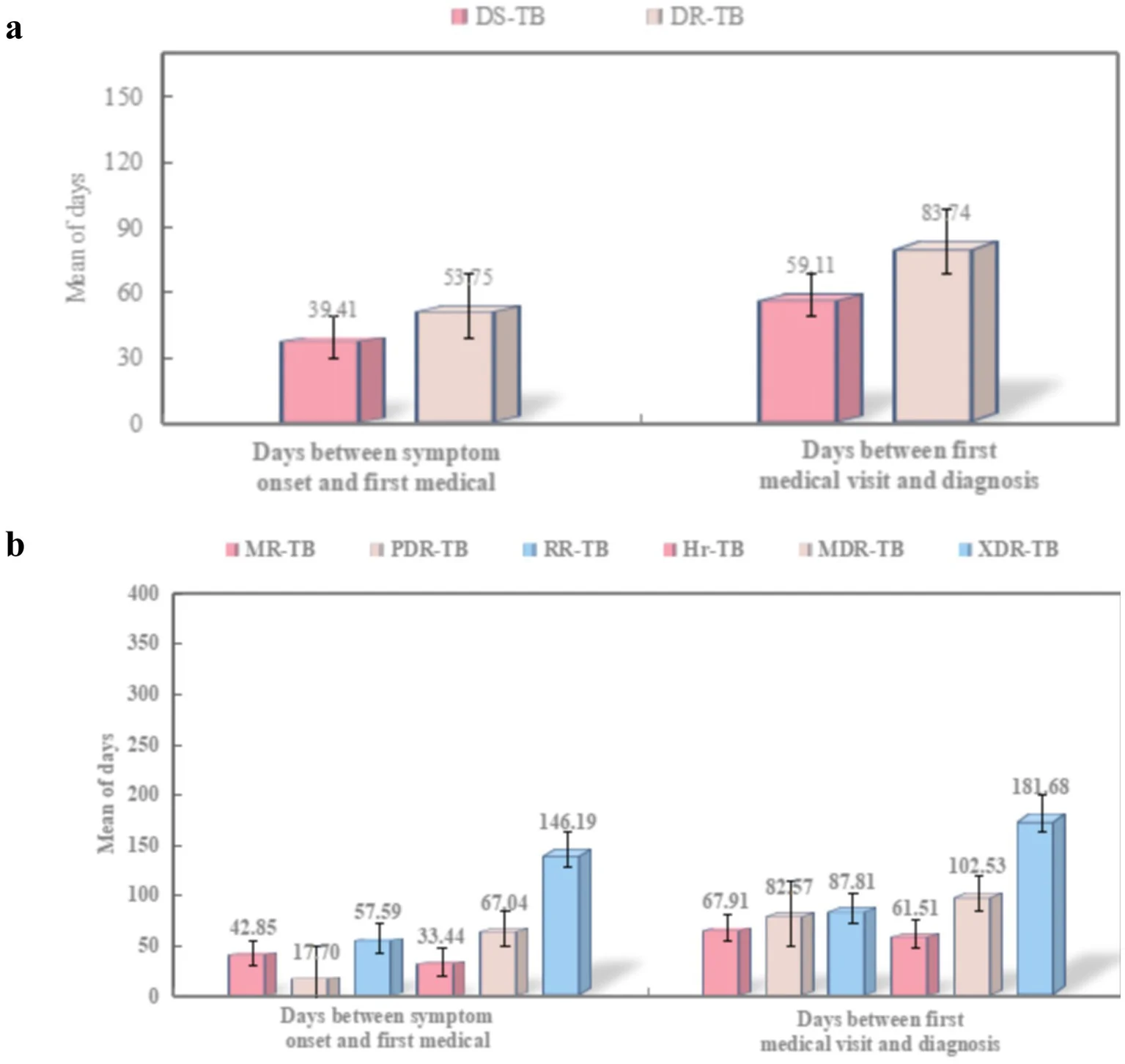
Diagnostic delays in tuberculosis (TB) patients. **(a)** Comparison of the mean duration (in days) between symptom onset and first medical visit, first visit and diagnosis for patients with DS-TB, DR-TB, **(b)** Comparison of the mean duration (in days) between symptom onset and first medical visit, first visit and diagnosis for patients with MR-TB, PDR-TB, RR-TB, Hr-TB, MDR-TB, and XDR-TB. TB, tuberculosis; DS-TB, drug-sensitive tuberculosis; DR-TB, drug-resistant tuberculosis; MR-TB, mono-resistant tuberculosis; PDR-TB, poly-resistant tuberculosis; RR-TB, rifampin resistance tuberculosis; Hr-TB, isoniazid resistance tuberculosis; MDR-TB, multidrug-resistant tuberculosis; XDR-TB, extensively drug-resistant tuberculosis.

Critically, DR-TB patients experienced significantly longer delays from symptom onset to first medical visit, and from first visit to diagnosis compared with DS-TB patients (*p* < 0.05) (Figure 3a). Among DR-TB subtypes, XDR-TB patients had the longest delays for both intervals (*p* < 0.05) (Figure 3b).

### 3.4 Multivariate logistic regression analysis

Multivariate logistic regression was performed to build predictive models and identify independent risk factors (see Table 2). Model 1 (Demographic): Significant risk factors included gender, age (<25, 25–44, 45–64), and occupation (unemployed, manual workers, business/service providers, office workers and students) (Supplementary Table 2). Model 2 (Clinical Symptom): HIV/AIDS comorbidity, cough and sputum production were associated with higher DR-TB risk, while hemoptysis was not significant (Supplementary Table 3). Model 3 (Diagnostic): At least one negative etiological test result (culture, smear, and molecular), previous TB treatment and concurrent EPTB were strong risk factors (Supplementary Table 4). Model 4 (Treatment Delay): Delays of 28–42 days between symptom onset to first visit and ≥42 days between the first visit to diagnosis, significantly increased DR-TB risk (Supplementary Table 5).

**Table 2:** Logistic regression analysis results of drug-resistant among TB patients in Shenzhen, 2017–2025.

Variables	Model 1				Model 2				Model 3				Model 4			
	*p*	OR	95% CI		*p*	OR	95% CI		*p*	OR	95% CI		*p*	OR	95% CI	
Gender (Female)	**0.009**	0.876	0.794	0.968	**0.013**	0.881	0.797	0.973	0.613	1.028	0.923	1.145	0.995	1.000	0.896	1.115
Age (<25)	1.000				1.000				1.000				1.000			
Age (25–44)	**0.003**	1.261	1.084	1.466	**0.013**	1.211	1.041	1.409	0.747	1.027	0.875	1.205	0.995	0.999	0.849	1.176
Age (45–64)	**0.003**	1.277	1.089	1.499	**0.060**	1.168	0.994	1.372	0.286	0.910	0.766	1.082	0.151	0.880	0.739	1.048
Age (≥65)	**0.009**	0.754	0.61	0.932	**0.000**	0.682	0.551	0.844	**0.000**	0.463	0.369	0.582	**0.000**	0.452	0.359	0.569
Occupation (Unemployed)	**1.000**				1.000				1.000				1.000			
Occupation (Family workers)	**0.018**	0.853	0.748	0.973	**0.017**	0.851	0.746	0.971	0.121	0.895	0.777	1.03	0.117	0.892	0.774	1.029
Occupation (Business service providers)	**0.000**	1.321	1.14	1.531	**0.012**	1.212	1.044	1.407	**0.000**	1.348	1.148	1.583	**0.000**	1.371	1.164	1.613
Occupation (Retirees)	0.159	0.840	0.659	1.071	0.087	0.808	0.633	1.031	0.331	0.878	0.676	1.141	0.356	0.883	0.678	1.15
Occupation (Office workers)	**0.002**	1.233	1.079	1.409	0.166	1.101	0.961	1.262	0.114	1.125	0.972	1.302	0.164	1.111	0.958	1.288
Occupation (Students)	**0.043**	0.751	0.569	0.991	**0.012**	0.701	0.530	0.926	0.033	0.726	0.541	0.975	**0.042**	0.735	0.546	0.989
Occupation (Others)	0.366	0.697	0.319	1.523	0.372	0.700	0.320	1.531	0.658	0.829	0.361	1.903	0.638	0.816	0.351	1.899
Combined with HIV/AIDS (Yes)					**0.034**	2.893	1.085	7.714	0.228	1.984	0.651	6.052	0.343	1.732	0.556	5.39
Cough (Yes)					**0.001**	1.242	1.088	1.418	0.162	1.106	0.96	1.274	0.139	1.114	0.965	1.286
Sputum production (Yes)					**0.000**	1.515	1.301	1.765	**0.001**	1.328	1.127	1.565	**0.001**	1.311	1.112	1.547
Hemoptysis (Yes)					0.113	1.171	0.963	1.423	0.135	0.850	0.687	1.052	0.878	0.983	0.793	1.219
Treatment type (Retreated patients)									**0.000**	7.232	6.421	8.144	**0.000**	7.033	6.232	7.938
Combined with EPTB infections (No)									**0.000**	0.672	0.594	0.761	**0.000**	0.661	0.584	0.749
Etiologic diagnosis results (≥1 negative in sputum culture, smear, or molecular test)									**0.000**	0.058	0.039	0.084	**0.000**	0.060	0.041	0.088
Days between symptom onset and first medical (**<**14)													1.000			
Days between symptom onset and first medical (14–28)													0.716	0.970	0.823	1.144
Days between symptom onset and first medical (28–42)													**0.003**	1.300	1.094	1.546
Days between symptom onset and first medical (≥42)													0.060	0.645	0.543	0.768
Days between first medical visit and diagnosis (**<**14)													1.000			
Days between first medical visit and diagnosis (14–28)													0.623	1.052	0.859	1.288
Days between first medical visit and diagnosis (28–42)													0.509	1.082	0.856	1.367
Days between first medical visit and diagnosis (≥42)													**0.000**	2.455	1.98	3.044
Constant	0.000	0.124			0.000	0.116			0.000	0.132			0.000	0.091		

EPTB, extra pulmonary tuberculosis; HIV/AIDS, Human immunodeficiency virus/Acquired immunodeficiency syndrome; OR, odds ratio; CI, confidence interval; *Model 1 adjusted for gender, age group, occupation. Model 2 adjusted for cough, sputum production, hemoptysis, and combined with HIV/AIDS at the base of Model 1. Model 3 adjusted for etiologic diagnosis results, treatment type and combined with EPTB infections at the base of Model 2. Model 4 adjusted for days between symptom onset and first medical, days between first medical visit and diagnosis at the base of Model 3. Bold values indicate statistically significant differences (*p* < 0.05).

### 3.5 Predictive performance of the models

**Figure 4:**
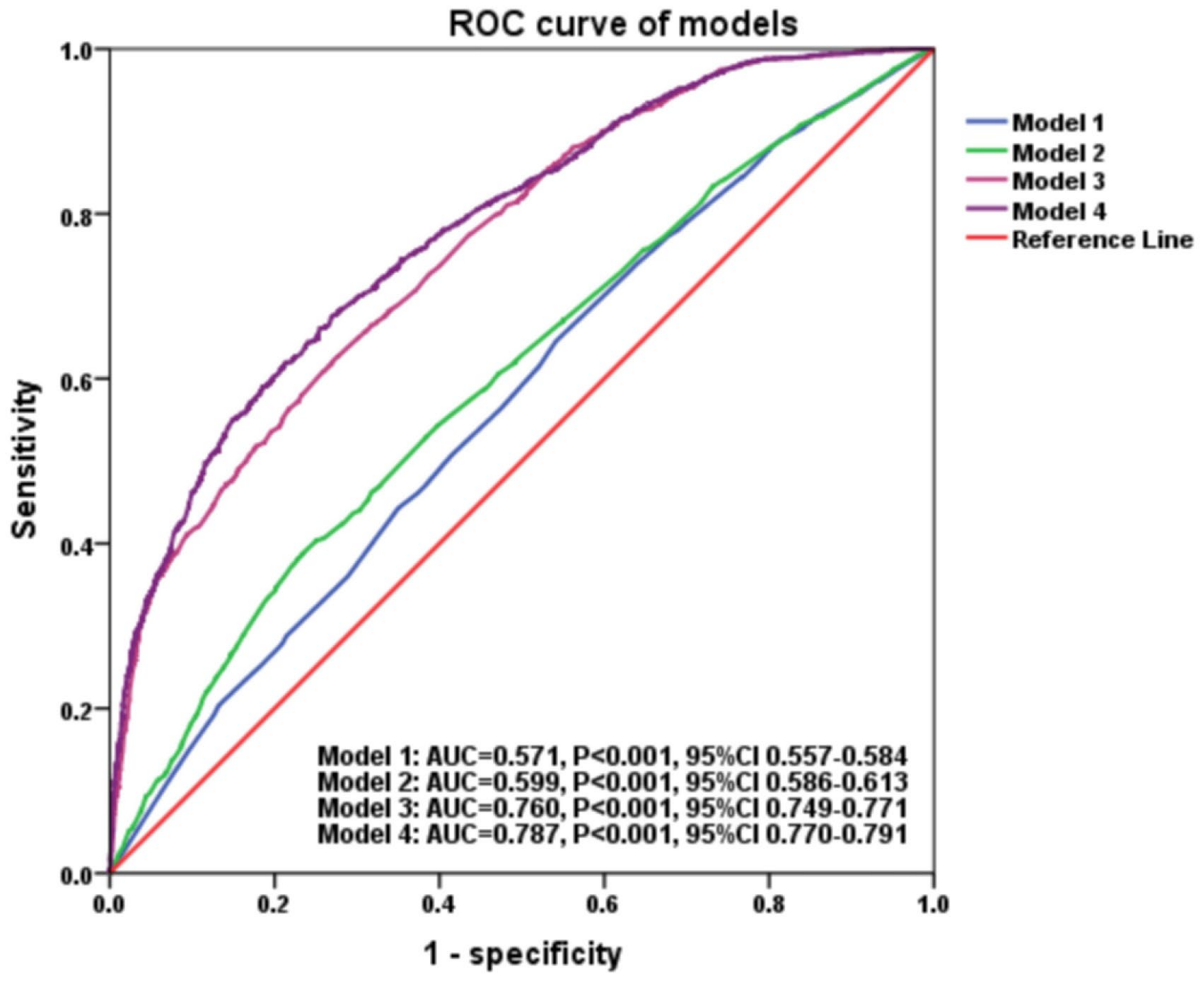
Predictive performance of the four drug-resistant tuberculosis (DR-TB) risk models. Receiver operating characteristic (ROC) curves for the four multivariate logistic regression models. Model 1: Area under the curve (AUC) = 0.571 (95% CI: 0.557–0.584), *p* < 0.001. Model 2: AUC = 0.599 (95% CI: 0.586–0.613), *p* < 0.001. Model 3: AUC = 0.760 (95% CI: 0.749–0.771), *p* < 0.001. Model 4: AUC = 0.787 (95% CI: 0.770–0.791), *p* < 0.001. AUC, area under curve; ROC, receiver operating characteristic; CI, confidence interval.

We evaluated discrimination using sensitivity, specificity, and ROC curves, and calibration using the Hosmer-Lemeshow test and calibration curves. The predictive performance of the four models is shown in Table 3. AUC values were 0.571 (95% CI 0.557–0.584), 0.599 (95% CI 0.586–0.613), 0.760 (95% CI 0.749–0.771) and 0.787 (95% CI 0.770–0.791) for Model 1–4, respectively (all *p* < 0.001), indicating that Model 4 had the highest discriminatory ability (Figure 4).

**Table 3:** Discrimination and calibration of four models.

Indicator	Discrimination				Calibration	
	Sensitivity	Specificity	AUC	95% CI	Hosmer-Lemeshow *χ*^2^	*p*
Model 1	0.645	0.459	0.571	0.557–0.584	14.014	0.081
Model 2	0.388	0.767	0.599	0.586–0.613	13.402	0.060
Model 3	0.518	0.823	0.760	0.749–0.771	9.618	0.293
Model 4	0.590	0.814	0.787	0.770–0.791	8.612	0.382

AUC, Area under the ROC curve; CI, confidence interval.

**Figure 5:**
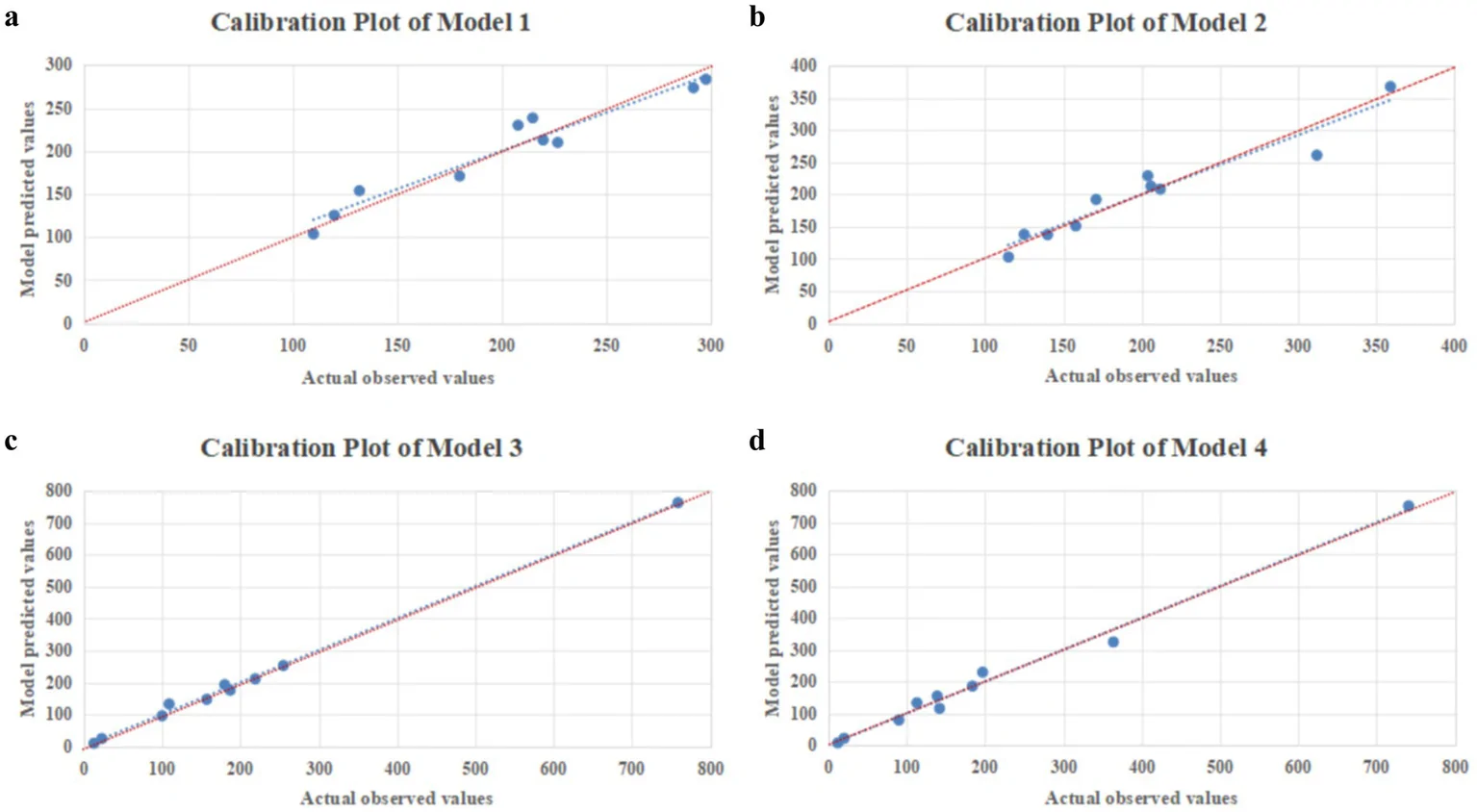
Calibration curves of four drug-resistant tuberculosis (DR-TB) risk models. **(a)** Calibration plot of Model 1. **(b)** Calibration plot of Model 2. **(c)** Calibration plot of Model 3. **(d)** Calibration plot of Model 4.

The Hosmer-Lemeshow χ^2^ values were 14.014, 13.402, 9.618 and 8.612, respectively (*p* > 0.05), indicating no significant difference between predicted and observed values. Calibration curves also showed that Model 4 had the best calibration accuracy (Figure 5).

## 4 Discussion

This study analyzed the epidemiological trends and risk factors for DR-TB in Shenzhen, China, from 2017 to 2025. As a major economic hub with a large migrant population, Shenzhen presents a unique context for TB epidemiology. Our data, drawn from the city’s primary designated DR-TB treatment center (Shenzhen Third People’s Hospital), provides a representative overview of the local situation.

We found that male patients had a higher risk of DR-TB than females. This may reflect higher mobility among men, who often work away from home, as well as gender differences in lifestyle (e.g., higher smoking and alcohol consumption) and health-seeking behaviors, which may lead to delayed treatment initiation.

Age was a significant risk factor, consistent with previous reports (12–14). Middle-aged adults (25–64 years), who are economically active and socially engaged, had a 1.2-fold higher risk of drug resistance than those under 25. This age group typically has greater social mobility, broader networks, higher occupational exposure, and thus greater susceptibility to DR-TB. Population mobility itself is a known risk factor for drug resistance (13). In China, incomplete management of the floating population, inadequate supervision of treatment adherence, and lack of social and medical security contribute to the emergence of DR-TB (14). As a region with a large floating population, Shenzhen urgently needs to strengthen diagnosis, treatment, and management for this group. The reduced risk among those aged ≥65 years may be due to lower social and occupational exposure, survival bias, underdiagnosis or different healthcare-seeking behavior (15–17).

The association between occupation and DR-TB risk appears complex and context-dependent. In our study, unemployed individuals and family workers – who have low mobility and stay at home for long periods – had lower infection risk (18). Students (aged 8–25 years) were mostly initial-treatment cases with relatively low resistance rates (19), possibly because of their higher education level, self-management skills, and good medication adherence (20). Business/service providers and office workers, on the other hand, have broad social contacts, more opportunities for TB exposure, and high work-related stress, which may impair immune function and treatment compliance, leading to higher resistance rates (21).

HIV/AIDS, a well-established comorbidity, was a significant risk factor in our models (13, 22–25). Research shows that TB patients with HIV/AIDS have a significantly higher risk of drug resistance (up to 19%) than HIV-negative patients (6%) (13). This is likely due to poorer drug tolerance and absorption, more severe side effects, lower cure rates, higher relapse risk, and immune impairment that promotes TB progression and transmission of resistant strains. However, the significance of HIV/AIDS disappeared after adjusting for treatment history, EPTB, and other factors, suggesting potential collinearity or limited power due to the small number of AIDS cases (n = 19). Similarly, diabetes was identified as a risk factor in some studies, but its effect was not stable across models. A history of previous TB treatment and retreatment were among the strongest risk factors for DR-TB (13, 16, 23, 26–29), underscoring the critical importance of effective initial treatment to prevent the resistance development.

Diagnostic factors played a significant role. As expected, positive sputum etiology was a risk factor (30, 31). Intriguingly, patients with positive sputum etiology had a vastly elevated risk, while those with negative result had a lower risk (OR ≤ 0.60). This suggests that inadequate diagnostic workup, rather than a specific negative result, is a major concern, potentially leading to missed DR-TB cases. This finding contrasts with a smaller study (29), possibly due to our larger sample size. Furthermore, delays between the first medical visit and diagnosis significantly increased DR-TB risk, underscoring the importance of reducing diagnostic confirmation delays.

Our analysis of clinical symptoms yielded novel insights. Cough and sputum production have been associated with DR-TB (30, 31), whereas hemoptysis were not. Hemoptysis is a classic symptom of pulmonary TB, but it is not specific for drug resistance. The link between hemoptysis and DR-TB has been rarely reported, and warrants further investigation. In our study, the lack of association may be due to differences in disease stage at diagnosis – hemoptysis often occurs in advanced cavitary disease, but many patients in our cohort were diagnosed earlier due to active screening (19). Furthermore, hemoptysis was recorded usually at the time of initial TB diagnosis (not at DR-TB confirmation), which could dilute the association.

The predictive power of our models increased as more variable categories were added, with Model 4 achieving the highest discrimination and calibration. The incremental AUC from Model 1 (0.571) to Model 4 (0.787), and that Model 3 (AUC 0.760) already performed well, indicating that demographic + clinical + diagnostic variables capture most predictive information. Compares our AUC with those of previous DR-TB prediction models (range 0.70–0.85) and notes that our model is within that range. In addition, an AUC increase of 0.03 from Model 3 to 4 is modest but statistically significant; however, the additional variables (Days between symptom onset and first medical, days between first medical visit and diagnosis) may not be available in all settings, so Model 3 may be more practical. This demonstrates the value of a multi-factorial approach for risk stratification, though the cost-effectiveness of implementing complex models in clinical practice must be considered.

This study has several limitations. Its single-center design, while ensuring data consistency, may limit generalizability across Shenzhen. Data integrity, particularly for laboratory results such as sputum cultures, may have been affected by the lengthy treatment process. The inclusion of numerous variables introduces potential collinearity and population heterogeneity. Finally, although the comprehensive model exhibited the best predictive performance, its feasibility and cost-effectiveness in routine settings-especially where data and resources are limited-remain to be determined. Future studies should assess cost-effectiveness of comprehensive predictive model.

## 5 Conclusion

We developed a robust predictive model for DR-TB based on 15 key risk factors, including demographic, clinical, diagnostic, and treatment history variables. These findings emphasize the need for heightened clinical vigilance and the early identification of at-risk individuals, particularly those with a history of TB treatment, diagnostic delays, and specific symptoms such as cough, and sputum production. Enhancing early diagnosis through targeted screening of high-risk groups is a critical step toward controlling DR-TB. Future research should validate these risk factors in multi-center studies and diverse populations to develop cost-effective, scalable screening strategies.

## Data Availability

The data analyzed in this study is subject to the following licenses/restrictions: Data was obtained from Shenzhen Third People’s Hospital, and are available from the author Xiangxiang Liu on reasonable request. Requests to access these datasets should be directed to Xiangxiang Liu, xxliu2014@126.com.
