# Supplementary material for: Risk factors and prediction modeling for drug-resistant tuberculosis in Shenzhen: a 9-year retrospective study
Source: Front Public Health. 2026 Sep 1;14:1875521. doi: 10.3389/fpubh.2026.1875521 (PMC13574663; doi:10.3389/fpubh.2026.1875521)
Supplement: Supplementary file 1 [file Data_Sheet_1.pdf]

Supplementary Table 1. Demographic and clinical characteristics of TB patients in Shenzhen, 2017–2025

Characteristic		DS-TB* (N=14464)		DR-TB * (N=2002)		Total TB* (N=16466)		x^2	*P-value*
		n	%	n	%	n	%		
**Gender**	Male	8910	61.60	1285	64.19	10195	61.92	4.982	**0.026**
	Female	5554	38.40	717	35.81	6271	38.08		
**Age**	＜25	2064	14.27	250	12.49	2314	14.05	52.195	**＜0.001**
	25-44	6319	43.69	946	47.25	7265	44.12		
	45-64	3963	27.40	619	30.92	4582	27.83		
	≥65	2118	14.64	187	9.34	2305	14.00		
**Mobility**	Intra-urban	5134	35.50	683	34.12	5817	35.33	3.382	0.184
	Inter-city	2537	17.54	335	16.73	2872	17.44		
	Inter provincial	6793	46.96	984	49.15	7777	47.23		
**Ethnicity**	Han	14270	98.66	1976	98.70	16246	98.66	0.024	0.876
	Others	194	1.34	26	1.30	220	1.34		
**Occupation**	Unemployed	4882	33.75	648	32.37	5530	33.58	63.366	**＜0.001**
	Family workers	3724	25.75	455	22.73	4179	25.38		
	Business service providers	1705	11.79	325	16.23	2030	12.33		
	Retirees	693	4.79	84	4.20	777	4.72		
	Office workers	2590	17.91	418	20.88	3008	18.27		
	Students	793	5.48	65	3.25	858	5.21		
	Others	77	0.53	7	0.35	84	0.51		
**Combined with HIV/AIDS**	No	14451	99.91	1996	99.70	16447	99.88	6.717	**0.010**
	Yes	13	0.09	6	0.30	19	0.21		
**Combined with Diabetes**	No	13212	91.34	1848	92.31	15060	91.46	2.091	0.148
	Yes	1252	8.66	154	7.69	1406	8.54		
**Close contacts of TB**	No	14457	99.95	2000	99.90	16457	99.95	0.854	0.355
	Yes	7	0.05	2	0.10	9	0.05		
**Cough**	No	10294	71.17	1217	60.79	11511	69.91	90.080	**＜0.001**
	Yes	4170	28.83	785	39.21	4955	30.09		
**Sputum production**	No	12417	85.85	1541	76.97	13958	84.77	107.272	**＜0.001**
	Yes	2047	14.15	461	23.03	2508	15.23		

**Fever**	No	13047	90.20	1782	89.01	14829	90.06	2.792	0.095
	Yes	1417	9.80	220	10.99	1637	9.94		
**Hemoptysis**	No	13753	95.08	1868	93.31	15621	94.87	11.415	**＜0.001**
	Yes	711	4.92	134	6.69	845	5.13		
**Chest pain**	No	13783	95.29	1889	94.36	15672	95.18	3.358	0.067
	Yes	681	4.71	113	5.64	794	4.82		
**Other clinical symptoms**	No	14267	98.64	1974	98.60	16241	98.63	0.033	0.855
	Yes	195	1.35	28	1.40	223	1.35		
**Etiologic diagnosis results**	Positive	11329	78.33	1974	98.60	13003	80.79	465.856	**＜0.001**
	≥1 negative in sputum culture, smear, or molecular test	3135	21.67	28	1.40	3163	19.21		
**Treatment type**	Newly treated patients	13446	92.96	1281	63.99	14727	89.44	1563.141	**＜0.001**
	Retreated patients	1018	7.04	721	36.01	1739	10.56		
**Combined with EPTB infections**	No	10076	69.66	1588	79.32	11664	70.84	79.406	**＜0.001**
	Yes	4388	30.34	414	20.68	4802	29.16		
**Days between symptom onset and first medical**	＜14	6472	44.75	724	36.16	7196	43.70	160.101	**＜0.001**
	14-28	2721	18.81	311	15.53	3032	18.41		
	28-42	1888	13.05	456	22.78	2344	14.24		
	≥42	3383	23.39	511	25.52	3894	23.65		
**Days between first medical visit and diagnosis**	＜14	2049	14.17	178	8.89	2227	13.52	242.323	**＜0.001**
	14-28	3900	26.96	360	17.98	4260	25.87		
	28-42	2551	17.64	273	13.64	2824	17.15		
	≥42	5964	41.23	1191	59.49	7155	43.45		
**Total**		14464	100.00	2002	100.00	16466	100.00		

TB, tuberculosis; DS-TB, drug-sensitive tuberculosis; DR-TB, drug-resistant tuberculosis; EPTB, extra pulmonary tuberculosis; HIV/AIDS, Human immunodeficiency virus/Acquired immunodeficiency syndrome.

Supplementary Table 2. Logistic regression analysis of drug-resistant among TB patients in model 1

Variables	*B*	*SE*	*Wals*	*P*	*Exp (B)*	*95% CI*	
**Gender (Female)**	-0.132	0.051	6.774	**0.009**	0.876	0.794	0.968
**Age (＜25)**			42.105	1.000			
**Age (25-44)**	0.232	0.077	9.051	**0.003**	1.261	1.084	1.466
**Age (45-64)**	0.245	0.082	9.001	**0.003**	1.277	1.089	1.499
**Age (≥65)**	-0.282	0.108	6.843	**0.009**	0.754	0.610	0.932
**Occupation (Unemployed)**			53.076	1.000			
**Occupation (Family workers)**	-0.159	0.067	5.618	**0.018**	0.853	0.748	0.973
**Occupation (Business service providers)**	0.278	0.075	13.735	**0.000**	1.321	1.140	1.531
**Occupation (Retirees)**	-0.175	0.124	1.983	0.159	0.840	0.659	1.071
**Occupation (Office workers)**	0.210	0.068	9.468	**0.002**	1.233	1.079	1.409
**Occupation (Students)**	-0.286	0.142	4.085	**0.043**	0.751	0.569	0.991
**Occupation (Others)**	-0.360	0.399	0.818	0.366	0.697	0.319	1.523
**Constant**	-2.092	0.082	648.850	0.000	0.124		

Model 1 adjusted for gender, age group, occupation.

Supplementary Table 3. Logistic regression analysis of drug-resistant among TB patients in model 2

Variables	*B*	*SE*	*Wals*	*P*	*Exp (B)*	*95% CI*	
**Gender (Female)**	-0.127	0.051	6.197	**0.013**	0.881	0.797	0.973
**Age (＜25)**			43.455	1.000			
**Age (25-44)**	0.192	0.077	6.169	**0.013**	1.211	1.041	1.409
**Age (45-64)**	0.155	0.082	3.544	0.060	1.168	0.994	1.372
**Age (≥65)**	-0.382	0.109	12.362	**0.000**	0.682	0.551	0.844
**Occupation (Unemployed)**			34.229	1.000			
**Occupation (Family workers)**	-0.161	0.067	5.728	**0.017**	0.851	0.746	0.971
**Occupation (Business service providers)**	0.192	0.076	6.375	**0.012**	1.212	1.044	1.407
**Occupation (Retirees)**	-0.213	0.124	2.936	0.087	0.808	0.633	1.031
**Occupation (Office workers)**	0.096	0.069	1.915	0.166	1.101	0.961	1.262
**Occupation (Students)**	-0.356	0.142	6.250	**0.012**	0.701	0.530	0.926
**Occupation (Others)**	-0.357	0.399	0.797	0.372	0.700	0.320	1.531
**Combined with HIV/AIDS (Yes)**	1.062	0.500	4.507	**0.034**	2.893	1.085	7.714
**Cough (Yes)**	0.217	0.068	10.331	**0.001**	1.242	1.088	1.418
**Sputum production (Yes)**	0.416	0.078	28.448	**0.000**	1.515	1.301	1.765
**Hemoptysis (Yes)**	0.158	0.100	2.513	0.113	1.171	0.963	1.423
**Constant**	-2.158	0.083	679.175	0.000	0.116		

Model 2 adjusted for gender, age group, occupation, combined with HIV/AIDS, cough, sputum production, hemoptysis.

Supplementary Table 4. Logistic regression analysis of drug-resistant among TB patients in model 3

Variables	*B*	*SE*	*Wals*	*P*	*Exp (B)*	*95% CI*	
**Gender (Female)**	0.028	0.055	0.256	0.613	1.028	0.923	1.145
**Age (＜25)**			65.676	1.000			
**Age (25-44)**	0.026	0.082	0.104	0.747	1.027	0.875	1.205
**Age (45-64)**	-0.094	0.088	1.139	0.286	0.910	0.766	1.082
**Age (≥65)**	-0.769	0.116	43.837	**0.000**	0.463	0.369	0.582
**Occupation (Unemployed)**			33.758	1.000			
**Occupation (Family workers)**	-0.111	0.072	2.403	0.121	0.895	0.777	1.030
**Occupation (Business service providers)**	0.299	0.082	13.306	**0.000**	1.348	1.148	1.583
**Occupation (Retirees)**	-0.130	0.133	0.946	0.331	0.878	0.676	1.141
**Occupation (Office workers)**	0.118	0.075	2.496	0.114	1.125	0.972	1.302
**Occupation (Students)**	-0.320	0.150	4.523	**0.033**	0.726	0.541	0.975
**Occupation (Others)**	-0.188	0.424	0.196	0.658	0.829	0.361	1.903
**Combined with HIV/AIDS (Yes)**	0.685	0.569	1.450	0.228	1.984	0.651	6.052
**Cough (Yes)**	0.101	0.072	1.957	0.162	1.106	0.960	1.274
**Sputum production (Yes)**	0.284	0.084	11.481	**0.00**1	1.328	1.127	1.565
**Hemoptysis (Yes)**	-0.162	0.109	2.234	0.135	0.850	0.687	1.052
**Treatment type (Retreated patients)**	1.978	0.061	1064.936	**0.000**	7.232	6.421	8.144
**Combined extra pulmonary tuberculosis infection (Yes)**	-0.397	0.063	39.763	**0.000**	0.672	0.594	0.761
**Etiologic diagnosis results (≥1 negative in sputum culture, smear, or molecular test)**	-2.854	0.193	218.504	**0.000**	0.058	0.039	0.084
**Constant**	-2.025	0.089	514.603	0.000	0.132		

Model 3 adjusted for gender, age group, occuption, combined with HIV/AIDS, cough, sputum production, hemoptysis, etiologic diagnosis results, treatment type and combined extra pulmonary tuberculosis infection.

Supplementary Table 5. Logistic regression analysis of drug-resistant among TB patients in model 4

Variables	*B*	*SE*	*Wals*	*P*	*Exp (B)*	*95% CI*	
**Gender (Female)**	0.000	0.056	0.000	0.995	1.000	0.896	1.115
**Age (＜25)**			64.929	1.000			
**Age (25-44)**	-0.001	0.083	0.000	0.995	0.999	0.849	1.176
**Age (45-64)**	-0.128	0.089	2.066	0.151	0.880	0.739	1.048
**Age (≥65)**	-0.793	0.118	45.603	**0.000**	0.452	0.359	0.569
**Occupation (Unemployed)**			34.029	1.000			
**Occupation (Family workers)**	-0.114	0.073	2.458	0.117	0.892	0.774	1.029
**Occupation (Business service providers)**	0.315	0.083	14.348	**0.000**	1.371	1.164	1.613
**Occupation (Retirees)**	-0.124	0.135	0.851	0.356	0.883	0.678	1.150
**Occupation (Office workers)**	0.105	0.075	1.933	0.164	1.111	0.958	1.288
**Occupation (Students)**	-0.308	0.151	4.145	0.042	0.735	0.546	0.989
**Occupation (Others)**	-0.203	0.431	0.222	0.638	0.816	0.351	1.899
**Combined with HIV/AIDS (Yes)**	0.549	0.579	0.898	0.343	1.732	0.556	5.390
**Cough (Yes)**	0.108	0.073	2.184	0.139	1.114	0.965	1.286
**Sputum production (Yes)**	0.271	0.084	10.325	**0.001**	1.311	1.112	1.547
**Hemoptysis (Yes)**	-0.017	0.110	0.024	0.878	0.983	0.793	1.219
**Treatment type (Retreated patients)**	1.951	0.062	998.629	**0.000**	7.033	6.232	7.938
**Combined extra pulmonary tuberculosis infection (Yes)**	-0.414	0.064	42.244	**0.000**	0.661	0.584	0.749
**Etiologic diagnosis results (≥1 negative in sputum culture, smear, or molecular test)**	-2.806	0.193	210.397	**0.000**	0.060	0.041	0.088
**Days between symptom onset and first medical (＜14)**			77.192	1.000			
**Days between symptom onset and first medical (14-28)**	-0.031	0.084	0.132	0.716	0.970	0.823	1.144
**Days between symptom onset and first medical (28-42)**	0.263	0.088	8.852	**0.003**	1.300	1.094	1.546
**Days between symptom onset and first medical (≥42)**	-0.438	0.089	24.447	**0.000**	0.645	0.543	0.768
**Days between first medical visit and diagnosis (＜14)**			146.317	1.000			
**Days between first medical visit and diagnosis (14-28)**	0.051	0.103	0.241	0.623	1.052	0.859	1.288
**Days between first medical visit and diagnosis (28-42)**	0.079	0.119	0.436	0.509	1.082	0.856	1.367
**Days between first medical visit and diagnosis (≥42)**	0.898	0.110	66.967	**0.000**	2.455	1.980	3.044
**Constant**	-2.398	0.117	421.121	0.000	0.091		

Model 4 adjusted for gender, age group, occupation, combined with HIV/AIDS, cough, sputum production, hemoptysis, etiologic diagnosis results, treatment type and combined extra pulmonary tuberculosis infection, days between symptom onset and first medical, and days between first medical visit and diagnosis.
